# Prevalence of intestinal parasites and associated factors among HIV patients receiving antiretroviral therapy at public hospitals in North Shoa Zone, Oromia, Ethiopia: Cross-sectional study

**DOI:** 10.1371/journal.pone.0357660

**Published:** 2026-09-01

**Authors:** Belay Merkeb Zewude, Wuletaw Tadesse Mekonnin, Desta Amensisa Tola, Negesse Bokona Rufe

**Affiliations:** Department of Medical Laboratory Sciences, College of Medical and Health Sciences, Salale University, Fitche, Ethiopia; Mekdela Amba University, ETHIOPIA

## Abstract

**Background:**

Intestinal parasite infections have a significant impact on HIV patients and are a serious global public health concern. In Ethiopia, the frequency of intestinal parasites among HIV patients is significant. The prevalence of intestinal parasites and associated factors among HIV-positive patients in the study area is unknown. Therefore, this study aims to determine the prevalence of intestinal parasites and associated factors among HIV positive patients receiving antiretroviral therapy (ART) at North Shoa public hospitals using direct wet mount, formol-ether concentration and modified Ziehl-Neelsen staining techniques.

**Methods:**

A cross-sectional study was conducted among HIV patients receiving ART at public hospitals in the North Shoa Zone, Oromia Region, Ethiopia, from February 2024 to May 2024. A total of 493 participants were included using systematic random sampling methods. Socio-demographic data and other associated factors were collected using a structured questionnaire. Stool specimens were collected in a clean container and examined for intestinal parasites. Data were analyzed using SPSS version 27. Bivariate and multivariable logistic regression analyses were performed to identify factors associated with intestinal parasitic infections.

**Results:**

In this study, 61.1% of participants resided in rural areas, with a mean age of 43.26 years (SD = ±12.38). The overall prevalence of intestinal parasitic infection among HIV patients in this study was 28% (95% CI: 24.1–31.8). Rural residence (AOR: 14.38; 95% CI: 5.52–37.47, P = 0.001), CD4 count <200 cells/µL (AOR: 7.08; 95% CI: 2.33–21.59, P = 0.001), and unprotected water source (AOR: 12.90; 95% CI: 3.01–55.36, P = 0.001) were significantly associated with intestinal parasitic infection.

**Conclusion:**

Intestinal parasitic infections were common among HIV/AIDS patients in North Shoa Zone hospitals. Rural residence, low CD4^+^ cell counts, and unprotected water sources were significantly associated with intestinal parasitic infections. Routine stool examination for patients with low CD4 + counts and improved access to safe drinking water, particularly in rural areas, are recommended. Further longitudinal studies with a comprehensive assessment of associated factors are recommended.

## Background

In developing countries, intestinal helminths and protozoan parasitic infections are among the most common, with a significant morbidity and mortality rate [1]. The severity of intestinal parasite infections depends on several factors, including the immunological status of the individuals [2].

Human immunodeficiency virus (HIV) depletes CD4 cells, weakening a person’s immunity and thereby predisposing them to opportunistic infections [3]. Co-infections with other infectious agents, such as intestinal parasites, are particularly concerning because HIV infection remains a global public health concern. The development of moderate to severe infections, which are often asymptomatic or mild in an immunocompetent host, is facilitated by the immunodeficiency linked to this virus [4].

Studies have suggested that *C. parvum*, *I.belli*, and *Blastocystis hominis* are common opportunistic parasites associated with AIDS illness [5]. Moreover, additional studies indicated that intestinal parasites were more prevalent in individuals with HIV than in those without HIV [6–8]. Immunosuppression (CD4 < 200 cells/µL) is more common in patients with intestinal parasitic infections than in patients without intestinal parasites [9].

According to the UNAIDS report, globally, 39 million people were living with HIV/AIDS in 2022 while 630,000 had died from AIDS-related illness. Eastern and southern Africa accounted for around 53.6% of the total cases [10].

Intestinal parasite infection is quite common in sub-Saharan Africa, where most HIV/AIDS patients reside [11]. A systematic review of a study conducted among HIV-positive patients reported a pooled prevalence of 39.15% of intestinal parasites [12]. This proportion is higher than the prevalence among the total population, which is 25.01% [13]. According to research findings, each person with HIV should be suspected of having an opportunistic intestinal parasite infection [14].

The diagnosis of parasitic infections relies on several laboratory methods. Microscopy is the primary tool for the detection of intestinal parasites. Intestinal parasites can be detected by a direct wet mount of stool specimens. However, when parasites are rarely found in direct wet mounts, concentration techniques are recommended to increase the likelihood of detection [15,16].

Due to inappropriate waste disposal, intestinal parasites are thought to be more prevalent among HIV-positive individuals in Ethiopia [17]. Despite these findings, most medical facilities lack sensitive diagnostic techniques to identify low levels of parasitic infection. Furthermore, the majority of healthcare facilities lack access to certain diagnostic techniques for particular intestinal parasites, particularly for some opportunistic intestinal parasites. The only technique for detecting intestinal parasites in North Shoa public hospital laboratories is the direct wet mount, which understates the true prevalence. Patients are not routinely tested for these pathogens at monitoring centers. Furthermore, there is a paucity of information regarding the prevalence of intestinal parasites and associated factors among HIV-positive patients in the study area. Thus, the objective of this study is to determine the prevalence of intestinal parasites and associated factors among HIV-positive patients receiving antiretroviral therapy (ART) at North Shoa public hospitals.

## Materials and methods

### Study design, period, and area

A hospital-based cross-sectional study was conducted in North Shoa Zone public hospitals, Oromia regional state, Ethiopia, from February 2024 to May 2024. There are 1,639,587 people living in 14 districts in this zone; 820,595 of them are men and 818,992 are women. According to the information obtained from the North Shoa Zone health bureau, there are 7 public hospitals, 63 health centers, and 267 health posts in the zone, which provide different health care services for the population. For this study, four hospitals (Salale University Comprehensive Specialized Hospital, Kuyu General Hospital, Chanco General Hospital, and Mukaturi Hospital) were selected using simple random sampling via a lottery system.

### Population

The source population for this study was all HIV patients receiving ART at public hospitals in the North Shoa Zone. The study population included all HIV patients receiving ART at the selected public hospitals in the North Shoa Zone during the study period.

### Eligibility criteria

All HIV patients receiving ART at selected public hospitals in the North Shoa Zone were included in the study, except for individuals who had taken anti-parasitic medications within the previous two weeks and who were severely ill at the time of data collection.

### Study variables

Dependent variable of the study was Intestinal parasitic infection status. The independent variables includes marital status, sex, educational status, age, address, source of drinking water, a habit of eating uncooked vegetables, a habit of hand washing existence of toilet, CD4 count, and WHO disease stage.

### Sample size calculation and sampling methods

The sample size was calculated using the sample size determination formula for a single population proportion. The sample size calculation considered a 73.3% proportion from the study conducted in Nekemte Specialized Hospital [7], along with a 95% confidence interval, a 5% margin of error, a design effect of 1.5, and an additional 10% to account for non-response. This led to a final sample size of 493 individuals.

To choose representative individuals, a multi-stage sampling procedure was used. First, a lottery was used to choose four hospitals at random: Salale University Comprehensive Specialized Hospital, Kuyu General Hospital, Chanco General Hospital, and Mukaturi Hospital. Each hospital received a proportionate share of the sample: 257 individuals were assigned to Salale University Comprehensive Specialized Hospital, 175 to Kuyu General Hospital, 35 to Chanco General Hospital, and 26 to Mukaturi Hospital. Lastly, systematic random sampling was used to choose eligible patients from each hospital.

### Measurement and data collection

#### Data collection procedure.

Socio-demographic characteristics, clinical presentation, treatment history, and other variables of the study participants were collected using a structured questionnaire after written informed consent/assent was obtained. Data collectors were hired, instructed, and trained to collect data using the pre-structured questionnaire.

Stool specimens were collected (4 g of formed stool and 10 mL of diarrheic stool) into wide-open-necked, carefully labeled, dry, leak-proof, and grease-free transparent stool containers by the participants. The participants were instructed on how to collect stool specimens at the point of care.

#### Laboratory analysis.

**Direct wet mount method:** Stool specimens were collected at the hospital laboratory and then tested for intestinal parasites using the direct wet mount method with normal saline (0.85% NaCl). To further enhance the detection of intestinal protozoan cysts, Lugol’s iodine staining was performed. The remaining stool specimens were preserved in 10% formalin for further analysis.

**Formol-ether concentration technique procedures:** The formol–ether concentration technique was performed for each sample. Approximately 1 gm of stool specimens were emulsified in 7 mL of 10% formol water contained in a screw-cap tube using an applicator stick. The emulsified samples were sieved, after which 3 mL of diethyl ether was added and the mixture was shaken vigorously and centrifuged at 2000 revolutions per minute for 3 minutes. After centrifugation, the sediment was transferred onto a slide, covered with a cover glass, and examined microscopically using 10× and 40 × objective lenses [18].

**Modified Ziehl–Neelsen method procedures:** A smear was prepared, air-dried, and fixed with methanol for 2–3 minutes. It was then stained with carbol fuchsin for 15 minutes, followed by decolorization with 1% acid alcohol for 15 seconds. The slide was counterstained with methylene blue for 30–60 seconds. Finally, the preparation was examined microscopically, using low power for detection and high power for identification [19].

### Data quality assurance

Throughout the whole research process, quality control procedures were put in place to guarantee the validity of the study’s conclusions. Prior to the start of the study, the questionnaire was pre-tested at Sendafa Hospital, training was given, and the principal investigator closely monitored the data collecting process.

Standard operating procedures were followed for all laboratory analyses. An adequate stool specimen (4 g of formed stool or 10 mL of diarrheic stool) was collected using carefully labeled, dry, leak-proof, and grease-free transparent stool containers. The specimens were kept free from water, soil, and urine contamination. Specimens contaminated with water, urine, or soil were rejected, and the study participants were requested to provide another sample. All reagents and supplies used to collect and analyze stool specimens were checked for quality. The stool examination was done independently by two qualified laboratory technicians. Direct stool examination was performed within 30 minutes to avoid delays. All microscopic findings and questionnaire-based information were properly coded and reported.

### Operational definitions

**Unprotected water source**: For this study, an unprotected water source was defined as a drinking-water source that was not adequately protected from contamination by human or animal waste or other environmental contaminants. This included water obtained from unprotected wells, rivers, streams, ponds, lakes, and other open surface-water sources.

### Data analysis and interpretation

Epidata version 4.6.0.2 was used to code and enter the data, which was then cleaned and exported to SPSS version 27.0 for analysis. Descriptive statistics were used to characterize the population. The association between socio-demographic characteristics and other risk factors of intestinal parasite infection was then investigated using bivariate and multivariable logistic regression models. To illustrate the strength of the association, the odds ratio at 95% CI was computed. A statistically significant p-value was considered less than 0.05.

The Spearman correlation coefficient and collinearity diagnoses, such as tolerance value > 0.1 and VIF (Variance Inflation Factor) < 10, were used to verify the logistic regression assumption. Once more, the Hosmer and Lemeshow p-value ≥ 0.05 and the Omnibus test of model p-value ≤ 0.05 were examined for goodness of fit.

### Ethical considerations

Ethical clearance was obtained from the Institutional Review Board (IRB) of Salale University (reference no. IHRPGn/188). A formal letter from Salale University was submitted to the selected hospitals to obtain permission to conduct the study. Written consent/assent was then obtained from all selected participants. The names of the study participants were omitted from the questionnaires, and code numbers were used instead to ensure confidentiality. The results of the study were communicated to the physicians so that the participants would receive the appropriate treatment.

## Result

### Demographic characteristics of study participants

A total of 493 ART-receiving participants were enrolled in this study. The participants’ ages ranged from 16 to 76 years, with a mean age of 43.26 (SD ± 12.38). Of the total participants, 251 (50.90%) were female and 242 (49.10%) were male. The majority of the participants (61.10%) were rural residents. Regarding occupation profile, 30.80% were private sector workers, 22.70% were government employees, 27.80% were farmers, and 17.80% were daily laborers. Approximately one-third 30.40% of the participants had no formal education. Regarding marital status, 51.50% were married, while the remaining were single, divorced, or widowed (Table 1).

**Table 1:** Socio-demographic characteristics of HIV-positive patients attending North Shoa Zone public hospitals.

Factors		Number of samples N	%
**Residence**	Urban	192	38.90%
	Rural	301	61.10%
**Sex**	Male	242	49.10%
	Female	251	50.90%
**Age**	11-20	2	0.40%
	21-30	80	16.20%
	31-40	154	31.2%
	41-50	128	26.00%
	>50	129	26.20%
**Marital status**	Single	70	14.20%
	Married	254	51.50%
	Divorced	45	9.10%
	Widowed	124	25.20%
**Educational status**	Illiterate	150	30.40%
	Read & write	136	27.60%
	Primary	92	18.70%
	Secondary	56	11.40%
	Above secondary	59	12.00%
**Occupation**	Daily labor	88	17.80%
	Employee	112	22.70%
	Private	152	30.80%
	Farmers	137	27.80%
	Students	4	0.80%

### Prevalence of intestinal parasites

Among the 493 participants, 28% were found to be infected with intestinal parasites. The predominant parasite was *Ascaris lumbricoides* (6.5%), followed by *Entamoeba histolytica dispar/moshkovskii* (5.9%), *Strongyloides stercoralis* (5.7%), and *Isospora belli* (0.2%). Double infection of *Entamoeba histolytica* dispar/moshkovskii and *Giardia lamblia* was also detected in 0.8% of the participants (Table 2).

**Table 2:** Prevalence of intestinal parasites among HIV-positive patients attending North Shoa Zone public hospitals.

Species	Frequency	%
*E. histolytica/dispar/moshkovskii*	29	5.9
*A. lumbricoides*	32	6.5
*I. belli*	1	0.2
*G. lamblia*	15	3
*H. nana*	11	2.2
*C. parvum*	9	1.8
*S. stercoralis*	28	5.7
*Taenia species*	9	1.8
Double Infection	4	0.8
Total	138	28.0

Nb: 138 (28.0%) represents single individual with at least one intestinal parasitic infection. Therefore the four patients with double infections were counted once in the overall total.

### Associated factors among HIV patients receiving ART

After controlling potential confounding variables, the multivariate logistic regression analysis identified three independent variables of intestinal parasitic infections among HIV patients. Significant associations were found with the following variables: rural residency (AOR: 14.38; 95% CI: 5.52–37.47, P = 0.001), CD4 count <200 cells/µL (AOR: 7.08; 95% CI: 2.33–21.59, P = 0.001), and unprotected water source (AOR: 12.90; 95% CI: 3.01–55.36, P = 0.001) (Table 3).

**Table 3:** Factors associated with intestinal parasitic infection among HIV-positive patients attending North Shoa Zone public hospitals.

Factors		Tested number	Positive	Positive (%)	COR (95%CI)	p- value	AOR(95%CI)	P-value
**Residence**	Urban	192	22	11.45%	1		1	
	Rural	301	116	38.54%	10.21 (4.40, 23.69)	0.001	14.38 (5.52, 37.47)	0.001
**HIV stage**	Stage I	151	24	15.9%	1			
	Stage II	125	13	10.40%	1.80 (0.19, 17.68)	0.973	0.891 (0.34, 2.12)	0.581
	Stage III	112	100	89.28%	4.96 (1.49, 15.00)	0.005	4.336 (0.99, 38.01)	0.082
	Stage IV	5	1	20.00%	1.087 (0.11, 11.22)	0.944	2.31 (0.45, 11.34)	0.202
**CD4 count**	>500	79	8	10.39%	1		1	
	200-500	172	24	13.95%	0.99 (0.49, 1.97)	0.090	4.087 (0.34, 21.59)	0.280
	<200	242	106	43.80%	4.957 (1.64, 15.00)	0.005	7.08 (2.33, 21.59)	0.001
**Sex**	Male	242	35	14.46%	1			
	Female	251	103	41.04%	4.45 (2.46, 8.04)	0.001	3.73 (0.82, 6.89)	0.305
**Hand washing**	No	99	40	40.40%	6.68 (3.16, 14.13)	0.001	5.41 (0.62, 11.19)	0.291
	Yes	394	98	24.87%	1			
**Water source**	Private pipe	151	15	9.93%	1		1	
	Public pipe	60	9	15.00%	1.03 (0.44, 2.41)	0.951	2.33 (.74, 5.14)	0.621
	Unprotected source	282	114	40.42%	14.70 (1.07, 34.65)	0.001	12.90 (3.01, 55.36)	0.001

## Discussion

In this study, the prevalence of intestinal parasite infections among HIV patients on antiretroviral therapy (ART) was reported to be 28% at 95% CI (24.1%–31.8%). This result agrees with reports from different regions of Ethiopia: University of Gondar Hospital (29.1%), Arba Minch Hospital (28.2%), and Debre Tabor General Hospital (25.3% [6,20,21]. The finding was also consistent with studies conducted in Colombia (29.2%) and the Eastern Cape (30%) [4,22]. This agreement could be due to similarity in study design, diagnostic techniques, study population, and economic status.

However, the overall prevalence reported in this study is lower than in studies from Debre Tabor General Hospital (31.7%), Mizan-Tepi University Teaching Hospital (31.5), Jimma Health Center (39.56%) and Nekemte Specialized Hospital (73.30%) [7,23–25]. Furthermore, the result of this study was lower than the pooled prevalence reported in Ethiopia [12]. Differences in ART duration, inclusion criteria, and geographical locations of the study sites may explain the observed variation. Studies that included newly diagnosed or severely immunosuppressed patients may have reported higher prevalence rates. Moreover, this study was conducted in a high-altitude area, which may be less favorable for the survival and transmission of many intestinal parasites.

It was, however, higher than studies done in Ghana (5.97%), Nigeria (20.9%), and Dessie Hospital (17.6%) [11,26,27]. Differences in sample size, lifestyle, sanitation habits, and access to clean drinking water could all account for these discrepancies. About 57% of participants in the current study reported they used water from unprotected sources.

Of the infected participants, 42% had protozoan infections and more than half, 58%, had helminthic parasite infections. 7.25% of the infected participants had opportunistic intestinal parasites, which are more common in immunocompromised patients. Furthermore, co-infections involving both *Giardia lamblia* and *Entamoeba histolytica dispar/moshkovskii* were identified in four individuals.

Regarding parasite species *Ascaris lumbricoides* was the most frequently detected helminth, with a prevalence of 6.5%. This finding was comparable to the study from Mizan-Tepi University Teaching Hospital (6.3%) [24]. However, it was slightly lower than the study from Nekemte Specialized Hospital (7.0%) [7]. The prevalence of *E.histolytica/dispar/moshkovskii* was 5.9%, which was lower than the study from Mizan-Tepi University Teaching Hospital (7.7%) and Nekemte Specialized Hospital (14.1%) [7,24]. The prevalence of *Strongyloides stercoralis* was 5.7%, which was higher than the study from Mizan-Tepi University Teaching Hospital (0.7%) and the study from Nekemte Specialized Hospital (3.8%) [7,24].

The prevalence of *Giardia lamblia* was 3.0%, which was lower than the report from Mizan-Tepi University Teaching Hospital (7.0%) and the report from Nekemte Specialized Hospital (27.0%) [7,24]. *Hymenolepis nana* prevalence was 2.2%, which was higher than 1.4% reported from Mizan-Tepi University Teaching Hospital [24], but similar to the study from Nekemte Specialized Hospital 2.2% [7]. Environmental conditions, hygiene practices, access to safe water, rural–urban composition, immune status, and difference in diagnostic methods might have been contributed for the observed variation in parasite species.

This study showed that HIV patients from rural areas had a higher rate of intestinal parasitic infections compared with urban residents (P < 0.05). This observation is similar to the findings reported in Kenya, Arba Minch, and Mizan Tepi [21,24,28], suggesting consistent rural–urban disparities in parasitic burden. Variations in access to sanitary facilities, safe water, and health services could account for the observed association. According to data from a recent Ethiopian meta-analysis, intestinal parasitic infections were five times more common among HIV patients without access to latrines than in those with sanitation facilities [12].

Rural communities have inadequate waste disposal infrastructure, rely on unprotected water sources, or engage in open defecation that facilitates feco-oral transmission of intestinal parasites [29]. HIV-positive people may also be more vulnerable due to a lack of routine parasitological screening services, delayed healthcare-seeking behavior, and restricted access to health education in rural locations [12,21]. Comparable environmental conditions, sanitary infrastructure, and socioeconomic factors across various locales may be the reason for the consistency of the result with the earlier research.

In this study, intestinal parasitic infections and CD4 counts < 200 cells/µL were significantly associated, suggesting that severe immunosuppression is still a significant predictor of opportunistic infections among HIV positive patients. CD4 plays a central role in mucosal immune defense by coordinating cell-mediated immune responses against intestinal pathogens. In advanced HIV infection, progressive CD4 cell depletion compromises intestinal immune surveillance, disrupts the integrity of the mucosal barrier, and lessens the host’s ability to eradicate parasitic infections, thereby increasing intestinal parasite susceptibility and persistence [30]. Because extreme immunodeficiency impairs the protective Th1 immune response necessary for efficient parasite clearance, those with CD4 counts <200 cells/µL are more susceptible to opportunistic parasitic infections [28,31]. This result is in line with research done in India, Debre Tabor, Mizan Tepi, Arba Minch, Northwest Ethiopia, and earlier systematic reviews in Ethiopia [20,21,24,30,32,33]. The association’s biological plausibility is strengthened by the consistency across investigations, which implies that CD4 count is a clinically significant predictor of intestinal parasite infection susceptibility as well as a laboratory indicator of HIV disease progression. Opportunistic intestinal parasites are more common in individuals with significant CD4 depletion because of compromised cellular immunity and decreased resistance to enteric infections, according to similar findings from previous research [34–36].

Opportunistic infections in people living with HIV are largely driven by a gradual weakening of the immune system, along with several clinical and social factors. As CD4 + T-cell levels decline, the body becomes less able to mount an effective immune response, making individuals more vulnerable to infections that would otherwise be controlled. This risk is especially high in patients with advanced stages of HIV disease. Beyond immune status, factors such as poor adherence to antiretroviral therapy (ART), malnutrition, and advanced WHO clinical stage also play an important role in increasing susceptibility to opportunistic infections among people receiving ART in Ethiopia. Evidence from recent systematic reviews and meta-analyses consistently shows that low CD4 counts, advanced disease stage, poor ART adherence, and under nutrition are all linked to a higher risk of opportunistic infections [37–39]. These findings align well with the results of the present study, where a CD4 count < 200 cells/µL was significantly associated with intestinal parasitic infection.

Source of water from unprotected water was another variable that was significantly associated with parasitic infections (P < 0.05) among HIV patients in this study. This study is in agreement with other studies conducted in Debre Tabor, Arba Minch, Borena, Butajira, and Northwest Ethiopia [20,21,30,40,41]. These results are consistent across several geographic contexts, indicating that contaminated water sources continue to be a common and enduring risk factor for HIV-positive people in Ethiopia.

Although WHO clinical stage III, gender, and handwashing practice were significantly associated with intestinal parasitic infection in the bivariable analysis, these associations were not retained in the multivariable model. This suggests that their apparent effects were influenced by confounding from other variables included in the analysis.

Generally, this study showed that intestinal parasitic infection is a public health problem among people living with HIV receiving ART. The burden was comparable to reports from several regions of Ethiopia and other low- and middle-income countries, although variations were observed across settings, likely due to differences in study design, immune status of participants, duration of ART, environmental conditions, and diagnostic approaches. Rural residence, use of unprotected water sources, and CD4 count < 200 cells/µL were significantly associated with infection, demonstrating the importance of cellular immunity in host defense.

### Limitations

This study has some limitations. Identification of *Entamoeba histolytica* was based on conventional microscopy, which cannot reliably distinguish it from *E. dispar* or *E. moshkovskii*; therefore, the reported prevalence should be interpreted with caution. In addition, microscopy-based diagnostic methods are less sensitive than molecular techniques for detecting certain intestinal parasites, and opportunistic parasites such as Microsporidia were not assessed because of limited laboratory resources, which may have underestimated the true burden of intestinal parasitic infections. Furthermore, the use of a single stool specimen may have reduced parasite detection because of the intermittent shedding of some parasites. These methodological limitations should be considered when interpreting the study findings.

## Conclusion and recommendation

In this study intestinal parasitic infections were found to be quite common among HIV/AIDS patients in North Shoa hospitals. Rural residence, low CD4^+^ cell counts, and not having access to safe drinking water were all significantly associated with intestinal parasitic infection. Therefore, as part of standard clinical follow-up, stool tests should be performed on HIV/AIDS patients with decreased CD4 + levels. Additionally, especially in rural areas, programs targeted at enhancing access to safe drinking water should be reinforced. Further longitudinal studies with a comprehensive assessment of associated factors are recommended.
